# Pelvic MRI findings in relapsed prostate cancer after radical prostatectomy

**DOI:** 10.1186/s13014-015-0574-6

**Published:** 2015-12-24

**Authors:** D. Hernandez, D. Salas, D. Giménez, P. Buitrago, S. Esquena, J. Palou, P. de la Torre, J. Pernas, I. Gich, G. Gómez de Segura, J. Craven-Bartle, G. Sancho

**Affiliations:** Department of Radiation Oncology, Hospital de la Santa Creu i Sant Pau, Universitat Autònoma de Barcelona, Sant Quintí, 89, 08041 Barcelona, Spain; Department of Radiology, Hospital de la Santa Creu i Sant Pau, Universitat Autònoma de Barcelona, Sant Quintí, 89, 08041 Barcelona, Spain; Department of Urology, Fundació Puigvert, Universitat Autònoma de Barcelona, Cartagena Str. 340-350, 08025 Barcelona, Spain; Department of Radiology, Fundació Puigvert, Universitat Autònoma de Barcelona, Cartagena Str. 340-350, 08025 Barcelona, Spain; Department of Clinical Epidemiology, Hospital de la Santa Creu i Sant Pau, Universitat Autònoma de Barcelona, Sant Quintí, 89, 08041 Barcelona, Spain

**Keywords:** Radical prostatectomy, Biochemical failure, Multiparametric MRI, Salvage radiotherapy

## Abstract

**Purpose/Objective:**

Little is known about the clinical impact of using multiparametric MRI to plan early salvage radiotherapy after radical prostatectomy. We aimed to evaluate the incidence and location of recurrence based on pelvic multiparametric MRI findings and to identify clinical variables predictive of positive imaging results.

**Materials and methods:**

We defined radiological criteria of local and lymph node malignancy and reviewed records and MRI studies of 70 patients with PSA recurrence after radical prostatectomy. We performed univariate and multivariate analysis to identify any association between clinical, pathological and treatment-related variables and imaging results.

**Results:**

Multiparametric MRI was positive in 33/70 patients. We found local and lymph node recurrence in 27 patients and 7 patients, respectively, with a median PSA value of 0.38 ng/ml. We found no statistically significant differences between patients with positive and negative multiparametric MRI for any variable. Shorter PSADT was associated with positive lymph nodes (median PSADT: 5.12 vs 12.70 months; p: 0.017).

**Conclusions:**

Nearly half the patients had visible disease in multiparametric MRI despite low PSA. Positive lymph nodes incidence should be considered when planning salvage radiotherapy, particularly in patients with a short PSADT.

## Introduction

Radical prostatectomy is the gold standard treatment for localized prostate cancer, but depending on the pathologic tumor stage, up to 60 % of patients who undergo radical prostatectomy develop biochemical recurrence and require further treatment [1]. Salvage radiotherapy is the only treatment with curative intention for these patients and has been associated with a three-fold increase in prostate cancer-specific survival when compared with observation [2]. Despite these good results, around 50 % patients have recurrence in the ten years after salvage radiotherapy [3]. Major factors contributing to the high risk of progression after radiotherapy are: 1) uncertainty about the best time to perform adjuvant or salvage radiotherapy [4]; 2) difficulties locating the site of recurrence; 3) the risk of missing subclinical disease when defining the clinical target volume (CTV); and 4) the possibility of delivering insufficient radiation dose.

**Fig. 1:**
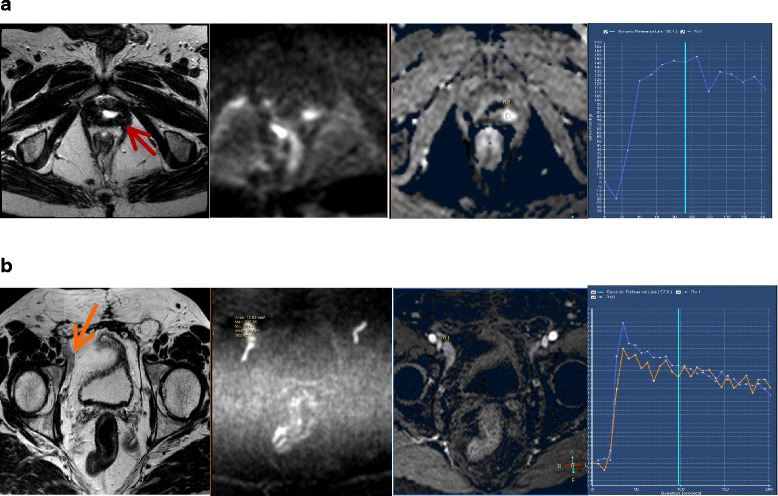
mpMRI malignancy criteria for local recurrence (**a**) and lymph node (**b**) recurrence. From left to right: T2-weighted images, DWI and dynamic contrast enhance image amb curve

Several studies [5–8] have investigated the accuracy of endorectal coil MRI for detecting local recurrence after radical prostatectomy. These studies mostly included men with mean PSA greater than 1 ng/ml and in many cases with clinically palpable recurrent disease. Moreover, none of these studies reported results combining three MRI techniques: T2-weighted imaging, dynamic contrast enhanced imaging and diffusion weighted imaging [8]. The main aim of this study was to evaluate the incidence and location of visible recurrence of prostate cancer on pelvic multiparametric MRI (mp-MRI) in men evaluated for early salvage radiotherapy. We also aimed to determine the association of clinical and pathological variables with the imaging results.

## Patients and methods

We performed a retrospective study in patients with prostate cancer who had biochemical failure after radical prostatectomy (PSA > 0.2 and increasing) and received salvage radiotherapy. Following our protocol, men referred for the consideration of salvage radiotherapy underwent an mp-MRI to investigate the site of recurrence before planning the radiation treatment. Patients without an mp-MRI study due to a clinical or technical contraindication were excluded. Surgical treatment was performed at the urology department at the hospital Fundación Puigvert and radiotherapy was carried out at the department of radiation oncology at the Hospital de la Santa Creu i Sant Pau. We pooled data from the clinical records of patients included in the study, and multidisciplinary review board approval was received from both hospitals participating in the study.

mp-MRI was performed on a 3-Tesla scanner (Achieva Medical Systems, Philips) using a surface phase array SENSE cardiac coil (six channels). MR imaging was acquired under the same conditions as the simulation CT: the patient in supine position with a knee support, empty rectum and comfortably full bladder.

The morphological study was obtained with T2-weighted turbo-spin echo (TSE) sequences in axial, coronal and sagittal planes using the following parameters: TR/TE 4278/100, 3 mm slice thickness, no gap and 300 FOV.

For functional evaluation, echo-planar diffusion sequences (DWI) TR/TE 2193/8, b values of 0, 1000, 1500 s/mm^2^, 4 mm slice thickness, matrix 140 × 120 were performed Apparent diffusion maps were generated and ADC (apparent diffusion coefficient values) were calculated by placing a region of interest (ROI) on the suspicious area.

Dynamic contrast enhance acquisition (DCE) was obtained by 3D gradient-echo-fat-suppressed sequence in the axial plane after injecting the paramagnetic contrast medium (0,1 ml/Kg Gadoteridol, Prohance, Bracco Amsterdam); 1,7 mm voxel size; matrix 148 × 146 and 7 mm dynamic scan time. MRI data processing was performed on a virtual work station (Intellispace Portal Phillips Systems V5.02.10011) by two uroradiologists with more than ten years’ experience, generating dynamic curves by placing a ROI in suspected areas. The radiologists were blinded to clinical and pathologic details but were aware of the biochemical failure status.

There is no validated scoring system to define a recurrence after radical prostatectomy on mp-MRI [5]. We used the PI-RADS [6] (Prostate Imaging Reporting and Data System for Prostate Cancer Detection with Multiparametric Magnetic Resonance Imaging) to define the radiological criteria of recurrence. Our mp-MRI malignancy criteria for local recurrence were: a soft tissue nodule in or around the prostatectomy bed in T2-weigthed images, DWI images based on a PI-RADS score between 3 and 5 and a DCE showing an early wash-in and type II and III curves based on PI-RADS score. We considered a recurrence when either DWI images or the DCE study showed abnormalities. Abnormalities exclusively on T2-weighted images were not indicative of a positive study. Lymph nodes were considered pathological when the short axis diameter was longer than 8 mm, the MRI signal was heterogeneous, the contour was irregular and the PI-RADS classification was 5, following the same criteria that are used for primary tumor (Fig. 1).

To investigate possible associations between clinical and pathological variables and imaging results, we recorded the initial PSA, pathological stage, pathological Gleason score, presence and location of extracapsular extension, vesicle involvement, margin status, number, morphology and size of positive margins, lymphatic and or vascular invasion, surgical modality (open, laparoscopy or robotic prostatectomy), postoperative PSA, nadir postoperative PSA, PSA at the time of MRI, and PSADT.

### Radiation treatment

Patients without visible recurrence in the mpMRI received salvage, three-dimensional conformal radiotherapy on pelvic lymph nodes (45 Gy) and prostatectomy bed (71 Gy). Patients with local recurrence received the same treatment plus a dose escalation on the tumor to 75 Gy. Patients with lymph node recurrence received salvage IMRT on pelvic lymph nodes (52.2 Gy in 29 fractions at 1.8 Gy/fraction) and the prostatectomy bed (63.80–65.25 Gy at 2.20–2.25 Gy/fraction) together with a simultaneous integrated boost (SIB) on the involved lymph nodes (60.9–62.35 in 29 fractions at 2.10–2.15 Gy/fraction). Androgen deprivation treatment was freely delivered based on the preferences of the attending physicians. No hormonal treatment was started before the mp-MRI was performed.

### Statistical analyses

We analysed differences in clinical, pathological and treatment-related variables between patients with positive and negative MRI studies using the T-test for continuous variables and the Chi-square test for categorical variables. Non-parametric tests (Mann-Whitney) were performed to test the medians of both populations. Logistic regression was performed for the multivariate analyses. Two-sided *P* values < 0.05 were considered statistically significant. Statistical analyses were performed using IBM-SPSS Inc, Chicago (USA) software for Windows (V22).

## Results

From June 2011 to June 2013, 300 patients with prostate cancer were referred to our department for radiation treatment. Of these, 70 patients were referred for salvage radiotherapy after radical prostatectomy and had undergone mp-MRI to investigate the site of recurrence.

The median time to diagnosis of recurrence was 38.8 months (2.39–171.50). Patient characteristics are shown in Table 1. The median age was 66 years (47–81). Most patients had pT2c or pT3 tumors and the Gleason score was greater than 7 in 32 % of patients. More than 70 % of the patients were treated with laparoscopy or robotic surgery and the neuro-vascular bundles were preserved in nearly half of them. Forty of 70 patients had positive surgical margins. Most of these positive margins were at the apex (17), followed by the right posterior prostate lobe (15). The mp-MR study showed visible disease in 33 out of 70 patients (47 %): local recurrence in 27 patients and lymph node recurrence in 7 patients (1 patient had both local and regional recurrence). We did not find statistically significant differences in clinical, pathologic and surgery modality variables between patients with positive and negative MR studies (Table 2).

**Table 1:** Clinical, pathologic and treatment related characteristics

	ALL (*n* = 70)
PSA (ng/ml) (Median)	7.8
Pathologic stage	
pT2a/b	11
pT2c	29
pT3a	20
pT3b	10
Lymph nodes (20)	
pN0	17
pN1	3
Gleason score	
≤ 7	48
> 7	22
Bladder neck involved (67)	
Yes	7
No	60
Lymphatic vessel invasion	
Yes	4
No	2
Unknown	64
Lymphadenectomy	
Yes	20
No	50
Radical prostatectomy	
Open	20
Laparoscopy/Robotic	50
Positive margin location	
Apex	17
Anterior right prostate lobe	7
Posterior right prostate lobe	15
Anterior left prostate lobe	4
Posterior left prostate lobe	7
Base	1

**Table 2:** Comparation of clinical, pathological and treatment related variables of patients with positive and negative MRI. Univariate analysis

	ALL	MR -	MR +	P*
	*n* = 70 (%)	*n* = 37 (%)	*n* = 33 (%)	
PSA (ng/ml) (median)	7.8	7.8	7.75	0.120
Pathologic stage				0.256
pT2	40 (57)	23 (33)	17 (24)	
pT3	30 (43)	14 (20)	16 (23)	
Lymph nodes (20)				0.215
pN0	17 (85)	12 (60)	5 (25)	
pN1	3 (15)	1 (5)	2 (10)	
Gleason score				0.527
≤ 7	48 (68)	25 (35)	23 (33)	
> 7	22 (32)	12 (17)	10 (15)	
Bladder neck involved (67)				0.382
Yes	7 (10)	3 (4)	4 (6)	
No	60 (90)	34 (51)	26 (39)	
Lymphatic vessel invasion				0.249
Yes	4 (6)	3 (4)	1 (2)	
No	2 (3)	2 (3)	0 (0)	
Unknown	64 (91)	32 (45.5)	32 (45.5)	
Positive margins				0.510
Yes	40 (57)	21 (30)	19 (27)	
No	30 (43)	16 (23)	14 (20)	
Lymphadenectomy				0.092
Yes	20 (29)	13 (19)	7 (10)	
No	50 (71)	24 (34)	26 (37)	
Radical prostatectomy				0.312
Open	20 (29)	12 (17)	8 (12)	
Laparoscopy/Robotic	50 (71)	25 (35.5)	25 (35.5)	
N-V bundle preservation				0.223
Yes	32 (46)	19 (27)	13 (19)	
No	38 (54)	18 (26)	20 (28)	
*PSA diagnostic (ng/ml)				0.630
Median	7.77 (3.98–58.63)	7.77 (3.98–58.63)	7.75 (4.1–32)	
*Nadir postop PSA (ng/ml)				0.101
Median	0.00 (0.00–6.68)	0.00 (0.00–0.70)	0.01 (0.00–6.68)	
*PSA at recurrence (ng/ml)				0.737
Median	0.38 (0.00–8.05)	0.41 (0.00–1.13)	0.37 (0.15–8.05)	
*PSADT (months)				0.446
Median	1.62 (0.67–153.24)	14.64 (0.67–49.95)	8.18 (2.48–145.2)	

*Median and range. Mann-Whitney Test. *pN1 (lymphadenectomy of 11 lymph nodes)

*IPSA:* PSA at diagnosis, *PSApRP:* PSA post radical prostatectomy, *PSApreRT:* PSA at the MRI, *PSADT:* PSA doubling time

### PSA values and kinetics

Median PSA at diagnosis, median nadir PSA after prostatectomy, and median PSA when the mp-MRI was performed were 7.8, 0.00 and 0.38 ng/ml respectively. The median PSADT was 11.26 months. No differences were found in PSA values and PSA kinetics between patients with positive and negative imaging studies (Table 2).

### Patients with local recurrence at the mp-MRI

Most local recurrences occurred at the perianastomotic site (19;27 %), followed by the right retrovesical (3;4.3 %), left retrovesical (1;1.5 %), periurethral (1;1.5 %), right seminal vesicle (1;1.5 %), left seminal vesicle (1;1.5 %) and penile bulb (1;1.5 %) sites. The median maximum diameter of the lesions was 8.5 mm (4.5–21 mm). We were unable to define a significant association between location of the local recurrence and any clinical, pathologic or surgery related variable.

### Patients with lymph node recurrence in the mp-MRI

mp-MRI detected 14 lymph node recurrences in 7 patients (10 %). The median time to diagnosis of recurrence was 24.3 months (range 2.8–75.4 months). The mean size of the pathological lymph nodes was 10 mm (range 8–16 mm). They were located in the right external iliac (5;7 %), the left external iliac (4;6 %), the right common iliac (1;1.5 %), the right internal iliac (1;1.5 %) and the right obturador regions (1;1.5 %). Lymph node recurrence showed radiological behaviour that was identical to local recurrence.

Five out of 7 patients with lymph node recurrence had a pT2c or pT3 tumor. The median PSA was 6.8 ng/ml (range: 5.45–10.98 ng/ml) before surgery. The median PSA was 0.45 ng/ml (range: 0.31–8.05 ng/ml) when the MRI was performed. The seven patients had a PSADT of less than 12 months. Patients with lymph node recurrence had a significantly shorter PSADT than those without recurrence (5.12 vs 12.70 months; (*p*: 0.017) (Table 3).

**Table 3:** PSA values and kinetics for patients with and without lymph node recurrence

	MR	Median	Range	*p*-value
PSA* (diagnosis)				0.628
	N+	6.80	5.17–25	
	N	8.12	3.98–58.63	
Nadir post-op PSA*				0.560
	N+	0.02	0.00–2.63	
	N	0.002	0.00–32.50	
PSA* (recurrence)				0.102
	N+	0.49	0.31–8.05	
	N	0.36	0.00–32.50	
PSADT**				0.017
	N+	5.12	2.58–11.52	
	N	12.70	1.98–153.24	

Median and range. Mann-Whitney Test; *ng/ml, ** PSA doubling time (months)

*N+* positive lymph nodes in the MRI, *N-* negative lymph nodes in the MRI

## Discussion

We aimed to evaluate the incidence and location of recurrence based on pelvic mp-MRI findings and to identify clinical variables predictive of positive imaging results. We found that the incidence of visible tumor in the mp-MRI was nearly 50 % despite a median PSA of 0.38 ng/ml. The incidence of local recurrence was 38 % (27/70 patients). Other authors [7–9] have reported an incidence ranging from 84 to 95 % using endorectal coil MRI. However, the patients in these series had a PSA higher than 1 ng/ml and larger lesions, some of which were clinically palpable by digital rectal examination. Studies on series of patients with a median PSA between 0.3 and 0.59 ng/ml, using endorectal coil MRI [5, 10] or pelvic coil [11–13], showed an incidence ranging from 24 to 73 %. These heterogeneous results could be due to the use of different technical protocols of imaging or to different imaging or pathologic criteria of local recurrence.

mp-MRI is the only imaging study recommended by the European Society of Urogenital Radiology (ESUR) to evaluate pelvic recurrences when the PSA is low (0.2–2 ng/ml) [6]. In men with PSA-recurrence below 0.5 ng/ml, CT and bone scan do not usually detect recurrence, and neither TRUS-guided biopsy of the perianastomotic region nor choline PET-CT have shown to accurately identify pelvis recurrence [14–17]. The results of a retrospective study [18] and one meta-analysis [19] have suggested a higher sensitivity of the choline PET-CT to identify cancer relapse after radical prostatectomy in patients with PSADT ≤ 6. Our incidence of nearly 50 % supports the ESUR recommendation [6] to use mp-MRI to evaluate men with biochemical failure prior to salvage radiotherapy.

Regarding location, our results are in line with earlier reports that found that most local recurrences occurred in the perianastomotic area and the retrovesical region [20]. Up to 22 % of recurrences have also been observed occurring at the resection of the vas deferens [21]. Currently, radiation oncologists plan salvage radiation treatment according to the published guidelines [20] to define the prostate bed and clinical target volume. Most patients receive blind salvage radiotherapy without investigating the exact site of recurrence because the benefit of planning individualised radiation treatment based on the results of MRI is still unknown. However, it has been suggested that the use of these guidelines misses part of the geographic recurrence in some cases [22, 23].

Few data are available on the ability of mp-MRI to detect lymph node recurrence, particularly, in patients with a slight increase of PSA after radical prostatectomy. In our series, despite the low PSA value, the mp-MRI detected 14 lymph node metastases in 7 patients (10 %), mainly in the external iliac region. Involvement of external iliac nodes could be related to the direct pathway of lymphatic drainage of the prostate along the vas deferens to the external iliac lymph nodes [21]. Liauw et al. [5] reported an incidence of lymph node recurrence of 5 %, but they used endorectal coil MRI with a different technical protocol. A recent study showed that diffusion-weighted MRI had an accuracy of 90 % for detecting lymph nodes smaller than 1 cm [24]. In two investigational studies [25, 26], other authors who performed MR lymphography with ferumoxtran-10 have reported an incidence of 72 and 20 % of positive lymph nodes of less than 1 cm in patients with biochemical recurrence after radical prostatectomy even when PSA was low. However, ferumoxtran-10 is only approved for clinical investigation. Despite these encouraging results, the sensitivity of the MRI and the ^11^C-choline PET/CT to detect lymph node metastases remains equally low [27, 28] even although it has been suggested to be slightly higher for diffusion-weighted MR [27].

The benefit of whole pelvis irradiation during salvage RT is controversial [29] but data from retrospective studies have shown a higher biochemical complete response rate [30] and biochemical relapse-free survival [31] when pelvic lymph nodes are included in the radiation field. One of the factors limiting the benefit of pelvic radiotherapy could be changes in the pattern of lymph node spread after radical prostatectomy. This different distribution of lymph nodes could not be adequately covered by the standard clinical target volume. In this setting, in one investigational study using ferumoxtran-10 MRI lymphography, 61 % of patients with PSA recurrence after radical prostatectomy, had positive lymph nodes outside the elective clinical target volume, despite a PSA lower than 1 ng/ml [25]. A recent retrospective study, that evaluated the usefulness of ^11^C-choline PET/TC in 605 patients with early recurrent prostate cancer referred for salvage radiotherapy, showed a higher incidence of pelvic lymph nodes when the PSADT was less than 6 months (17.1 vs 2.1 %) [18]. The incidence of 10 % in our study indicates lymph node recurrence should be kept in mind when planning salvage radiotherapy, even when PSA is below 1 ng/ml.

Several points of interest arose when we analysed the association between clinical factors and mp-MRI findings. When we compared patients with and without lymph node recurrence in the mp-MRI separately, we observed a low PSA at recurrence and a statistically significant shorter PSADT (median 5.12 vs 12.7 months; p: 0.017) . These results are in line with other authors who investigated lymph node spread on MRI lymphography using ferumoxtran-10 in patients with biochemical failure after RP (PSADT: 3.86 months) [25]. On the other hand, Couñago et al.,[12], showed a higher probability of radiographic local relapse when the PASDT was over 14 months. It would be interesting to investigate the clinical impact of the lymph node irradiation in patients who have a short PSADT without positive lymph nodes in the mp-MRI. Alternatively, it could be advisable to irradiate only the lymph node areas in patients with positive lymph recurrence in the mp-MRI and no visible tumor in the prostate bed or high-risk factors of local recurrence.

Our findings regarding pathologic positive margins differ from those reported by Verma et al. who observed that radiological recurrence was three times more likely when surgical margins were involved by tumor. Although we observed an association between the PSA at recurrence and MRI findings with a trend towards a significance, we were unable to define a PSA cut-off as a predictor of positive MRI. Other authors, however , have reported a pre-RT cut-off value of >0.3 [5], >0.5 [12] and ≥ 0.54 ng/ml [11] as a predictor of positive DCE-MRI.

Our study has several limitations. It was a retrospective analysis of a relatively small number of patients and the radiologists were not totally blinded to clinical information. Our results are based exclusively on radiological findings and we lack histological confirmation of local or nodal recurrences. Nevertheless, radiological and pathological correlations have been established in treatment-naive patients with prostate cancer [24]. The strengths of this study are that all mp-MRI were performed at the same department and were all analysed by two expert uroradiologists who defined the radiology criteria of malignancy before starting the review of the studies.

In view of the results, we have slightly modified our current imaging protocol. We have widened pelvic size for the DCE-MRI and DW sequences to ensure the functional study encompass lymph nodes in the highest region of the pelvis. These patients have been treated under a protocol of dose escalation on visible recurrence which will allow us to analyse its clinical impact.

## Conclusion

Our results support the use of mp-MRI to plan salvage radiotherapy after radical prostatectomy, even in patients with low PSA levels. Positive lymph nodes incidence should be considered, particularly in patients with a short PSADT.

## References

[1] Pfister D, Bolla M, Briganti A, Carroll P, Cozzarini C, Joniau S (2014). Early salvage radiotherapy following radical prostatectomy. Eur Urol.

[2] Trock BJ, Han M, Freedland SJ, DeWeese TL, Partin AW, Walsh PC (2008). Prostate cancer-specific survival following salvage radiotherapy vs observation in men with biochemical recurrence after radical prostatectomy. JAMA.

[3] Stephenson AJ, Scardino PT, Kattan MW, Pisansky TM, Slawin KM, Klein EA (2007). Predicting the outcome of salvage radiation therapy for recurrent prostate cancer after radical prostatectomy. J Clin Oncol..

[4] Siegmann A, Bottke D, Faehndrich J, Brachert M, Lohm G, Miller K (2012). Salvage radiotherapy after prostatectomy - what is the best time to treat?. Radiother Oncol.

[5] Liauw S, Pitroda S, Eggener S, Stadler WM, Pelizzari CA, Vannier MW (2013). Evaluation of the prostate bed for local recurrence after radical prostatectomy using endorectal magnetic resonance imaging. Int J Radiat Oncol Biol Phys.

[6] Barentsz JO, Richenberg J, Clements R, Choyke P, Verma S, Villeirs G (2012). ESUR prostate MR guidelines 2012. Eur Radiol..

[7] Sella T, Schwart LH, Swindle PW, Onyebuchi CN, Scardino PT (2004). Scher Hl, et al. Suspected local recurrence after radical prostatectomy: endorectal coil MR imaging. Radiology.

[8] Cirillo S, Petracchini M, Scotti L, Gallo T, Macer A, Bona MC (2009). Endorectal magnetic resonance imaging at 1.5 Tesla to asses local recurrence following radical prostatectomy using T2-weighted and contrast-enhanced imaging. Eur Radiol.

[9] Barchetti F, Panebianco V (2014). Multiparametric MRI for recurrent prostate cancer post radical prostatectomy and postradiation therapy. Biomed Res Int.

[10] Linder BJ, Kawashima A, Woodrum DA, Tollefson MK, Karnes J, Davis BJ (2014). Early localization of recurrent prostate cancer after prostatectomy by endorectal coil magnetic resonance imaging. Can J Urol.

[11] Rischke HC, Schäfer AO, Nestle U, Volegova-Neher N, Henne K, Benz MR (2012). Detection of local recurrent prostate cancer after radical prostatectomy in terms of salvage radiotherapy using dynamic contrast enhanced-MRI without endorectal coil. Radiat Oncol..

[12] Couñago F, Cerro ED, Recio M, Diaz AA, Marcos FJ, Cerezo L (2015). Role of 3T multiparametric magnetic resonance imaging without endorectal coil in the detection of local recurrret prostate cáncer after radical prosttectomy: the radiation oncology point of view. Scand J Urol..

[13] Verma V, Chen L, Michalski JM, Hu Y, Zhang W, Robinson K (2015). Evaluation of 3 T pelvic MRI imaging in prostate cáncer patients receiving post-prostatectomy IMRT. World J Urol.

[14] European Association of Urology. Guidelines on prostate cancer 2015. Update March 2015. https://uroweb.org/guidelines/.

[15] Giovacchini G, Picchio M, Coradeschi E, Bettinardi V, Gianolli L, Scattoni V (2010). Predictive factors of [(11)C]choline PET/CT in patients with biochemical failure after radical prostatectomy. Eur J Nucl Med Mol Imaging..

[16] Kitajima K, Murphy RC, Nathan MA, Froemming AT, Hagen CE, Takahashi N (2014). Detection of recurrent prostate cancer after radical prostatectomy: comparison of 11C-choline PET/CT with pelvic multiparametric MR imaging with endorectal coil. J Nucl Med.

[17] Panebianco V, Sciarra A, Lisi D, Lisi D, Galati F, Buonocore V (2012). Prostate cancer: 1HMRS-DCEMR at 3 T versus [(18)F]choline PET/CT in the detection of local prostate cancer recurrence in men with biochemical progression after radical retropubic prostatectomy (RRP). Eur J Radiol..

[18] Castellucci P, Ceci F, Graziani F, Schiavina R, Brunocilla E, Mazzarotto R (2014). Early biochemical relapse after radical prostatectomy: which prostate cancer patients may benefit from a restaging ^11^C-Choline PET/TC scan before salvage radiation therapy. J Nucl Med.

[19] Treglia G, Ceriani L, Sadeghi R, Ciovacchini G, Giovanella L (2014). Relationship between prostate-specific antigen kinetics and detection rate of radiolabelled choline PET/CT in restaging prostate cancer patients: a meta-analysis. Clin Chem Lab Med.

[20] Poortmans P, Bossi A, Vandeputte K, Bosset M, Miralbell R, Maingon P (2007). Guidelines for target volume definition in post-operative radiotherapy for prostate cancer, on behalf of the EORTC Radiation Oncologuy Group. Radiother Oncol.

[21] Nguyen DP, Giannarini G, Seiler R, Schiller R, Thoeny HC, Thalmann GN (2013). Local recurrence after retropubic radical prostatectomy for prostate cancer does not exclusively occur at the anastomotic site. BJU Int.

[22] Croke J, Malone S, Roustan N, Belanger E, Avruch L, Morash C (2012). Postoperative radiotherapy in prostate cancer: the case of the missing target. Int J Radiat Oncol Biol Phys.

[23] Wang L, Kudchadker R, Choi S, Pettaway CA, Choi H, Hobbs BD (2014). MD Local recurrence map to guide target volume delineation after radical prostatectomy. Pract Rad Oncol.

[24] Thoeny HC, Froehlich JM, Triantafyllou M, Huesler J, Bains LK, Vermathen P (2014). Metastases in normal-sized pelvic lymph nodes: detection with diffusion-weighted MR imaging. Radiology..

[25] Meijer HJM, Van Lin EN, Debats OA, Witjes JA, Span PN, Kaanders JH (2012). High occurrence of aberrant lymph node spread on magnetic resonance lymphography in prostate cancer ’patients with a biochemical recurrence after radical prostatectomy. Int J Radiat Oncol Biol Phys.

[26] Heesakkers RAM, Jager GJ, Hövels AM, de Hoop B, van den Bosch HC, Raat F (2009). Prostate cancer: detection of lymph node metastases outside the routine surgical area with ferumostran-10-enhanced MR imaging. Radiology.

[27] Budiharto T, Joniau S, Lerut E, Van den Bergh L, Mottaghy F, Deroose CM (2011). Prospective evaluation of 11C-choline positron emission tomography/computed tomography and diffusion-weighted magnetic resonance imaging for the nodal staging of prostate cancer with a high risk of lymph node metastases. Eur Urol.

[28] Heck MM, Souvatzoglou M, Retz M, Nawroth R, Kübler H, Maurer T (2014). Prospective comparison of computed tomography, diffusion-weighted magnetic resonance imaging and [11C]choline positrón emission tomography/computed tomography for preoperative lymph node staging in prostate cáncer patients. Eur J Nucl Mol Imaging..

[29] Alongi F, Fiorino C, Cozzarini C, Broggi S, Perna L, Cattaneo GM (2009). IMRT significantly reduces acute toxicity of whole-pelvis irradiation in patients treated with post-operative adjuvant or salvage radiotherapy after radical prostatectomy. Radiother Oncol.

[30] Moghanaki D, Koontz BF, Karlin JD, Wan W, Mukhopadhay N, Hagan MP (2013). Elective irradiation od pelvic lymph nodes during postprostatectomy salvage radiotherapy. Cancer.

[31] Spiotto MT, Hancock SL, King CR (2007). Radiotherapy after prostatectomy: improved biochemical relapse-free survival with wholw pwlvic compared with prostate bed only for high-risk patients. Int J Radiat Oncol Biol Phys.

